# Gas Separation Properties of Polyimide Thin Films on Ceramic Supports for High Temperature Applications

**DOI:** 10.3390/membranes8010016

**Published:** 2018-03-07

**Authors:** Sara Escorihuela, Alberto Tena, Sergey Shishatskiy, Sonia Escolástico, Torsten Brinkmann, Jose Manuel Serra, Volker Abetz

**Affiliations:** 1 Instituto de Tecnología Química (UPV-CSIC), Universitat Politècnica de València, Avda. Los Naranjos, s/n, 46022 Valencia, Spain; saesro@itq.upv.es (S.E.); soesro@upvnet.upv.es (S.E.); 2 Helmholtz-Zentrum Geesthacht, Institute of Polymer Research, Max-Planck-Str.1, 21502 Geesthacht, Germany; sergey.shishatskiy@hzg.de (S.S.); torsten.brinkmann@hzg.de (T.B.); 3 University of Hamburg, Institute of Physical Chemistry, Grindelallee 117, 20146 Hamburg, Germany

**Keywords:** Polymer/Ceramic Thin Film Composite Membrane, entanglement concentration, hydrogen, carbon dioxide, gas separation membranes, intrinsic viscosity, polyimides

## Abstract

Novel selective ceramic-supported thin polyimide films produced in a single dip coating step are proposed for membrane applications at elevated temperatures. Layers of the polyimides P84^®^, Matrimid 5218^®^, and 6FDA-6FpDA were successfully deposited onto porous alumina supports. In order to tackle the poor compatibility between ceramic support and polymer, and to get defect-free thin films, the effect of the viscosity of the polymer solution was studied, giving the entanglement concentration (C*) for each polymer. The C* values were 3.09 wt. % for the 6FDA-6FpDA, 3.52 wt. % for Matrimid^®^, and 4.30 wt. % for P84^®^. A minimum polymer solution concentration necessary for defect-free film formation was found for each polymer, with the inverse order to the intrinsic viscosities (P84^®^ ≥ Matrimid^®^ >> 6FDA-6FpDA). The effect of the temperature on the permeance of prepared membranes was studied for H_2_, CH_4_, N_2_, O_2_, and CO_2_. As expected, activation energy of permeance for hydrogen was higher than for CO_2_, resulting in H_2_/CO_2_ selectivity increase with temperature. More densely packed polymers lead to materials that are more selective at elevated temperatures.

## 1. Introduction

The existing socioeconomic situation creates an increase of the energy demand in both industrialized and developing nations, expected to be doubled by 2050 [1]. This makes the usage of highly-valuable energetic resources, the minimization of emissions of contaminant gases to the atmosphere, and the search for more efficient technologies for energy production at moderate costs, mandatory. Natural gas (mainly formed by CH_4_ and a small amount of higher alkanes and alkenes) is considered as one of the most promising fossil energy sources because it possesses the largest heat of combustion relative to the amount of CO_2_ formed (low carbon footprint). The valorization of methane from an indirect conversion outstands as the best option for the use of natural gas [2]. This approach requires synthesis gas (syngas) as an intermediate product for the process of converting methane into valuable chemicals.

Hydrogen production is the largest use of syngas, with steam methane reforming (SMR) being the predominant technology, which is a well-established process with two main reactions, reforming and water gas shift reaction [3,4]. In order to use the hydrogen obtained by this method, an extra purification step is necessary. Apart from hydrogen, carbon dioxide is mainly generated during the process (15–20%) among other gases [5]. In this sense, gas separation using membranes has been extensively investigated as one of the most energy efficient separation processes, and as a strong alternative to conventional purification systems.

Different types of membranes for hydrogen separation have been developed, and they can be classified according to the selective layer material as polymeric, metallic, ceramic, and carbon membranes [5]. Inorganic membranes have been widely studied for different industrial applications, due to their high temperature stability and their durability in harsh environments [6,7,8,9]. Membrane processes have shown to be beneficial, thanks to the process intensification that enables them to reach high energy efficiency in the plant, high yield per pass and high selectivity–lower yield to waste byproducts. However, the main disadvantage of inorganic membranes is related to economic and fabrication issues [10]. On the other hand, polymeric membranes show advantageous properties, such as ease of processing and low cost, and they would be more accessible for large-scale applications over inorganic membranes. Unfortunately, polymeric membranes have a strong limitation regarding high operating temperatures and mechanical stability [11]. In order to overcome these problems, polymeric materials that can operate at temperatures above 250 °C and exhibit rigid structures are needed. 

Separation of hydrogen from other small molecules, such as e.g., gases, requires rigid structures with voids, which form molecular transport channels, able to discriminate permeating molecules by size. In the case of polymers, this consideration limits the choice of selective materials to glassy polymers, where permeability selectivity is governed by the diffusion selectivity, and not by the solubility selectivity [12]. Permeability factors of gases in glassy polymers are highly dependent on the fractional free volume (FFV), which can be defined as the free space that is not occupied by the polymer molecules [13]. According to the solution-diffusion theory of molecular transport in polymers, the permeability (P) coefficient is determined by two factors: solubility (S), related to the properties of the gas and to its interaction with the polymer matrix; and diffusivity (D), which depends mainly on the ability of the gas molecules to move through the bulk of a polymer by migrating from one free volume void to another. At elevated temperatures, as in the case of several industrial processes, the solubility factor is even more reduced, and the diffusivity factor is more dominant compared to ambient temperatures. Therefore, under those conditions, the permeability selectivity will be mainly influenced by the differences in the diffusion coefficients of gases dissolved in the polymer. Polyimides, as high-performance materials, present high chemical and thermal stability and high glass-transition temperature [14]. Therefore, these materials can be employed in high temperature applications, such as the water-gas shift process.

For practical applications, the polymer membranes have to be processed as thin film composite membranes (TFCMs), where the supporting structures of the selective layer should possess high thermal and chemical stability whilst being highly permeable at the same time. Therefore, in this work, a high thermally resistant ceramic porous support and a dense layer of a highly stable polymer are combined. However, it is hard to achieve good compatibility between polymers and ceramic materials. One typical mismatch of properties of these materials origins from the method of polymer layer deposition on the porous ceramic by application of a polymer solution. The inorganic surface is not changing its properties upon the contact to organic solvent, while polymer undergo a drastic change of its state from diluted solution via mechanically weak gel to solid state. This change of the polymer state is accompanied by significant stress within the formed polymer layer, and on the polymer/ceramic interface. In order to obtain defect free polymer layer, it is obligatory to have ceramic support surface of a very high quality, without any defects with sharp edges, which can cause defect formation during polymer film drying [15]. 

The study of the coating conditions is crucial in order to get a good, defect free selective layer on top of the inorganic porous support, which will ensure reproducible experimental results. The effect of the concentration of the polymer solution for three different polyimides on the properties of deposited thin polymeric layers on top of an alumina support is studied. To overcome the challenge of polymer/inorganic incompatibility, the deposition of the polymer layer was studied in dependence of the polymer solution concentration and, consequently, the polymer solution viscosity. A number of commercial and in-house synthesized polymers with outstanding gas transport properties for several gas pairs, such as CO_2_/CH_4_ or O_2_/N_2_, were chosen for coating experiments, in order to study the effect of polymer composition on the properties of TFCMs. The gas transport properties of the obtained membranes were studied with a variety of gases as a function of temperature.

## 2. Experimental Section

### 2.1. Materials

Monomers for polyimide synthesis are 4,4′-(hexafluoroisopropylidene) diphthalic anhydride (6FDA) and 2,2-bis(4-aminophenyl) hexafluoropropane (6FpDA), which were purchased from Sigma-Aldrich. Polyimides P84^®^and Matrimid^®^ 5218 were purchased from HP Polymer GmbH (Austria) and Huntsman, respectively. Reactants and solvents, such as chlorotrimethylsilane (CTMS), pyridine (Py), acetic anhydride, *N*,*N*-dimethylaminopyridine (DMAP), *o*-xylene, anhydrous *N*-methyl-2-pyrrolidone (NMP), tetrahydrofuran (THF), dimethyl sulfoxide (DMSO), and dimethyl acetamide (DMAc) of reagent grade quality were all purchased from Sigma-Aldrich, and were used without further purification.

**Figure 1:**
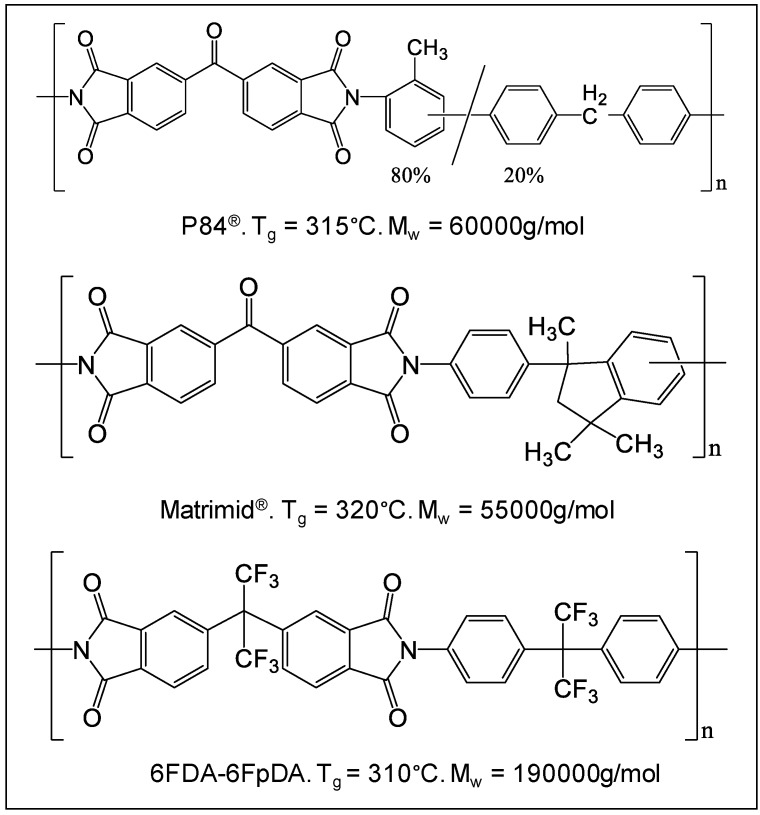
Structures and properties of the polyimides used in this work.

Three different polyimides were selected attending the criteria mentioned above, commercial P84^®^ and Matrimid^®^, and 6FDA-6FpDA polyimide synthesized for the current work. All these polymers possess high thermal stability, chemical resistance, and mechanical properties suitable for the chosen application. Figure 1 shows the glass transition temperatures (T_g_), and the molecular weight (M_w_) of these polyimides, as well as their chemical structures.

The 6FDA-6FpDA polyimide was synthesized following the classical in situ silylation two-step method [16]. A three-necked flask, equipped with a mechanical stirrer and gas inlet and outlet, was charged with 5.0 mmol of the diamine 6FpDA and 5.0 mL of solvent (NMP). The solution was stirred at room temperature under argon atmosphere until the solid was completely dissolved. Then, the solution was cooled, by the use of an ice bath, to 0 °C, and the required amount of CTMS and Py (1 mol/mol reactive group) and small amounts of DMAP (0.1 mol/mol Py) were added to the mixture. At that moment, the temperature was raised up to room temperature to ensure the formation of the silylated diamine. 

After this, the corresponding dianhydride 6FDA (5.0 mmol) and additional solvent were added. The reaction mixture was left overnight to ensure the formation of the corresponding poly(amic acid) in the solution. The viscosity of the solution significantly increased during this period. Afterwards, the reaction was completed by chemical imidization. For this purpose, an excess of acetic anhydride (20.0 mmol) and Py (10.0 mmol) was added to the poly(amic acid) solutions; the obtained mixture was heated to 60 °C, and stirred vigorously for 8 h. Afterwards, the mixture was precipitated in distilled water and repeatedly washed in a water/ethanol mixture. The polymer was dried under vacuum at 120 °C for 24 h.

Regarding the ceramic supports, Whatman^®^ Anodisc inorganic filter was purchased from Sigma-Aldrich, with a diameter of 25 mm and pore size of 0.02 µm [17]. Anodisc was used as support material because of its high purity alumina matrix that combines good thermal stability, solvent compatibility, and very regular porous structure. It is bonded to a polypropylene ring at the outer circumference for ease of handling and coating. 

Polymer solutions were prepared using THF as a solvent for Matrimid^®^ and 6FDA-6FpDA polymers, and NMP in the case of P84^®^. Several concentrations were prepared (0.5–1–2.5–3–4–5–7.5–8–10 wt. %) in order to detect the optimal concentration for each polymer. 

**Figure 2:**
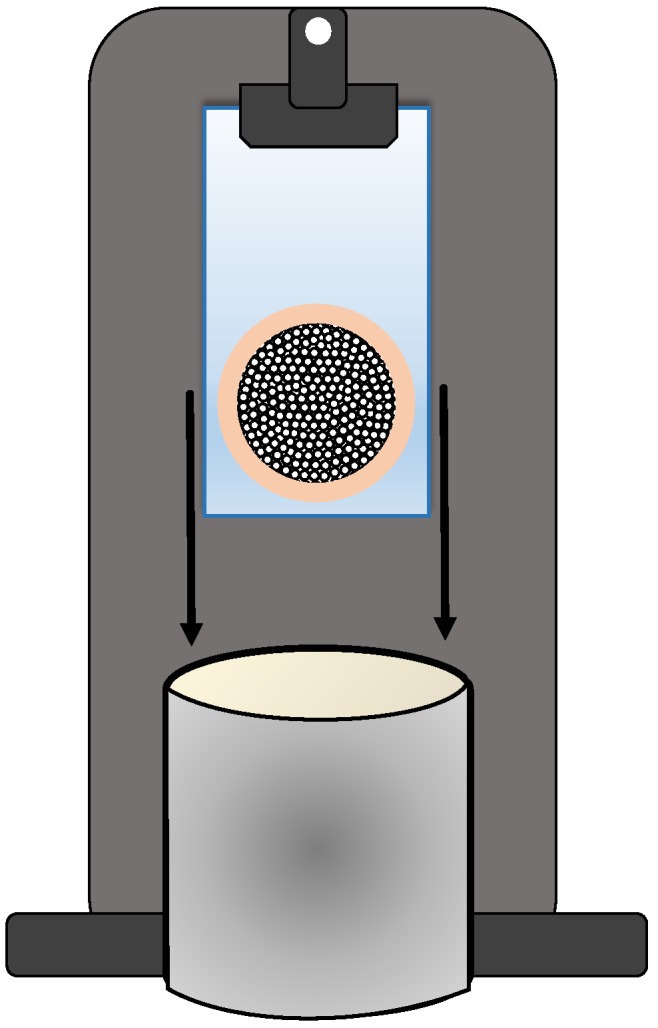
Schematics to the dip coating configuration and process.

Anodisc supports were placed on top of a glass plate and fixed with Kapton adhesive tape. Additionally, the Anodisc supports were covered with more Kapton tape at the bottom and outer edges, in order to ensure that the polymer is only placed on top of the porous membrane. In this way, only one side of the Anodisc membrane was in direct contact with the polymer solution. The supports prepared in this way were then dip-coated with the corresponding polymer solution at 250 mm/min speed, perpendicular orientation to the solution (Figure 2), and with no waiting downtime. The correct deposition of the polymer on top of the Anodisc support was confirmed visually when the membranes were removed from the glass plate. It was also checked that the part of the glass plate below the membrane was dry. In addition, the correct deposition of the polymeric layer was checked by FE-SEM (Carl Zeiss Microscopy GmbH, Oberkochen, Germany) as well. Temperature and relative humidity were controlled and kept constant, in order to avoid defects during the coating, such as pinholes or non-continuous layer formation. 

### 2.2. Characterization 

Thermogravimetric analysis (TGA) was used to evaluate the thermal stability of the polymers. Disc-shaped samples, cut from cast films, with weights between 5 and 15 mg were tested. TGA experiments were performed on a Thermal Analysis NETZSCH TG209 F1 Iris instrument (Gerätebau GmbH, Selb, Germany). The experiments were accomplished under a flow of 20 mL/min of argon in the temperature range from 25 to 800 °C, with a heating rate of 5 K/min.

Differential scanning calorimetry (DSC) analysis was used to determine the T_g_ of polymers. DSC experiments were carried out with a calorimeter DSC 1 (Mettler Toledo, Greifensee, Switzerland), within the temperature range from 50 °C to 450 °C, at a heating rate of 10 K/min. Measurements were accomplished in nitrogen atmosphere to prevent oxidation. Usually, the glass transition is determined in the second heating cycle to avoid the effect of sample preparation history rising, for example, from the remaining solvent traces.

The apparent molecular weight of the copolymers was determined by gel permeation chromatography (GPC) after calibration with polystyrene standards. GPC measurements were performed at 40 °C, having DMAc as eluent on a Waters instrument (Waters GmbH, Eschborn, Germany) equipped with polystyrene gel columns of different pore sizes, using a refractive index (RI) detector. 

The fractional free volume (FFV) was determined from density measurements. A density determination kit (Mettler Toledo, Greifensee, Switzerland) was employed. The auxiliary liquid for the measurement was isooctane as a liquid with low density and very low solubility in studied polymers. FFV was calculated according to the method described elsewhere [18]. 

The effective viscosity was measured using a Lovis 2000 M/ME Microviscometer (Anton Paar GmbH, Graz, Austria). Different solution concentrations of the three different polymers were investigated in order to obtain a relation between solution concentration and solution viscosity. Accuracy of the equipment is 0.5% and 0.05 °C for the temperature. 

The single gas permeances of prepared TFCMs were determined by using the “pressure increase” facility, utilizing the “constant volume, variable pressure” method [19]. Single-gas permeation data were determined at 1000 mbar feed pressure and in the temperature range from 30 °C to 90 °C (maximum operating temperature of the equipment is around 100 °C). The “pressure increase” facility was described in detail elsewhere [20]. Briefly, the facility determines the rate of a pressure increase in the calibrated permeate volume when certain feed pressure is applied to the membrane of a certain area. The facility is connected to 15 gas lines, and each gas can be taken into the feed volume of the facility. The measurement part of the facility is thermostated with 0.1 °C precision, and all valves and pressure sensors have no heating elements. The facility can be programmed to carry out gas transport experiments with any gas connected to it, at any feed pressure in the range 100–1200 mbar, and permeate pressure 0–13 mbar with a temperature step 1 °C and higher [21]. 

The permeability coefficient P [cm^3^ (STP) cm cm^−2^ s^−1^ cmHg^−1^] of single gas was determined as

(1) $$L=\frac{V_{p}\;l\;22.4}{R\;T\;At}\ln\left(\frac{p_{f}-p_{0}}{p_{f}-p_{P\left(t\right)}}\right)$$

where $V_{p}$ is the constant permeate volume (cm^3^), l is the film thickness (cm), A is the effective area of membrane (cm^2^), R is the gas constant (8.314 J mol^−1^ K^−1^), t is the time (s) for permeate pressure increase from *p_0_*, to *p_P_*_(*t*)_, $p_{f}$ is the feed pressure (cmHg).

The diffusion coefficient D (cm^2^ s^−1^) was calculated from membrane thickness l (cm) and time lag θ (s) determined graphically as intersection of the line drawn through the linear region of the pressure increase curve to intersection with the time axis, as it can be seen in Equation (2).

(2) $$D=\frac{l^{2}}{6\theta }$$

The solubility coefficient S (cm^3^ (STP) cm^−3^ cmHg^−1^) was calculated according to the following equation:

(3) $$S=\frac{P}{D}$$

The ideal selectivity ($\alpha_{A/B}$) of the material can be expressed as the ratio of permeability coefficients of two penetrants and, according to Equation (3), it is a function of diffusion and solubility selectivity, leading to the equation:

(4) $$\alpha_{A/B}=\frac{P_{A}}{P_{B}}=\frac{D_{A}S_{A}}{D_{B}S_{B}}$$

The single gas permeance *L* (m^3^ (STP) m^−2^h^−1^ bar) of the membrane can be calculated using the equation similar to the Equation (1):

(5) $$L=\frac{V_{p}\;22.4}{R\;T\;At}\ln\left(\frac{p_{f}-p_{0}}{p_{f}-p_{P\left(t\right)}}\right)$$

where *V_P_* is the constant permeate volume (m^3^), *R* is the gas constant (8.314 J mol^−1^ K^−1^), *A* is the effective area of membrane (m^2^), *t* (h) is the time of measurement and *p_f_, p_0_*, and *p_P_*_(*t*)_ (bar) are pressures at the feed, permeate side at the beginning, and permeate side at the end of the measurement, respectively. The factor of 22.4 is used to transfer from molar to volumetric units (assuming ideal gas behavior).

Field-emission scanning electron microscope (FE-SEM) (Zeiss Ultra 55), equipped with EDX for elemental analysis was used for measuring the thickness of the samples and studying the contact quality of organic/inorganic materials, as well as the correct and the continuous formation of thin film layers.

## 3. Results and Discussion

The TGA showed the thermal stability of the polymers studied in this work. In all the cases, a single weight loss step was observed at temperatures higher than 450 °C. This is an indication of the high thermal stability of these polymers, and provides an idea about the possible working temperature range. Thermal glass transition (T_g_) for these polymers was determined by DSC, and it was found that all three polymers have T_g_ very close to each other, with Matrimid^®^ having the highest: Matrimid^®^ (320 °C) > P84^®^ (315 °C) > 6FDA-6FpDA (310 °C).

As was mentioned above, the separation properties are strongly influenced by the FFV of the polymers [22]. Table 1 shows the permeability coefficients and ideal selectivity of the three polymers studied as a thick film for different gases. 6FDA-6FpDA presents the highest permeability, followed by Matrimid^®^, and P84^®^. In the case of 6FDA-6FpDA, the values were experimentally obtained, whereas from the other two polymers, values were found in literature [23,24].

All the membranes were fabricated by a single coating step. The possibility to dip-coat more than once (multiple steps), in order to avoid the possible pinholes, as in previous studies [25], was considered. However, this technique was dismissed, due to the better reproducibility and homogeneity of one-step dip-coating, and also to prevent the chance of massive polymer penetration into pores of the porous support. 

**Figure 3:**
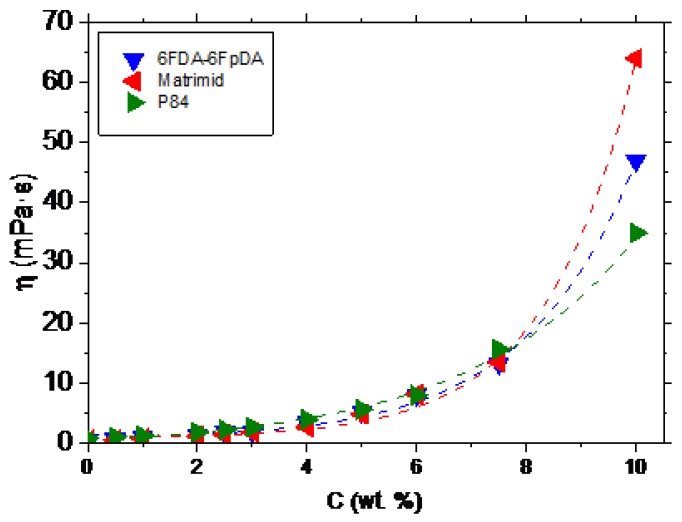
Evolution of the viscosity as a function of the polymer concentration (dotted lines are eye guides only).

The relation between the polymer concentration and the viscosity (*η*) was studied. This relation allows to establish the minimum viscosity needed to form a continuous defect-free layer on top of the ceramic support. The evolution of the viscosity as a function of the polymer concentration, for 6FDA-6FpDA, Matrimid^®^, and P84^®^, is depicted in Figure 3. The polymers showed an exponential increase of the viscosity with the polymer concentration, as was described for other polymeric systems [26,27,28]. 

In a dilute solution, the polymer coils have enough space to avoid any interaction. The viscoelasticity in a dilute solution is therefore attributable to the properties of individual polymer coils. The overall viscoelasticity exhibited by a dilute solution is the viscoelasticity associated with each coil, multiplied by the number of coils present. This behavior is described by the Rouse–Zimm model [29]. If the concentration of polymer coils is increased beyond certain limit, the coils can no longer remain discrete, and become prone to entanglement.

Table 2 describes the nomenclature of the functional forms of viscosities employed in the calculations described below.

The viscosities of solutions of various concentrations can be modelled by Huggins and Kraemer equations [30,31,32]. Huggins equation is defined as

(6) $$\frac{\eta_{sp}}{C}=\eta +k_{H}{\left[\eta \right]}^{2}C$$

where *k_H_* is denominated Huggins constant and has values ranging from 0.3 in good solvents to 0.5 in poor solvents. *k_H_* contains information about hydrodynamic and thermodynamic interactions between coils in solution. A plot of the reduced viscosity, extrapolated to zero concentration, yields the intrinsic viscosity. Using the expression for the specific viscosity in the Huggins equation above, Equation (6) provides Equation (7), the Kraemer equation.

(7) $$\eta_{red}=1+\left[\eta \right]C+k_{H}{\left[\eta \right]}^{2}C^{2}$$

**Figure 4:**
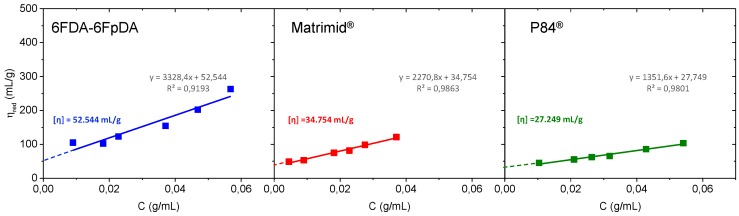
Huggins representation for intrinsic viscosity determination.

For a range of moderate concentrations, Huggins equation exhibits a linear dependence, while Kramer plot is linear only at sufficiently low concentrations. In the present study, the solution concentrations are considered as moderate concentrations. Consequently, Figure 4 shows the Huggins plot, where the intercept of the line at the ordinate corresponds to the intrinsic viscosity ([η]).

**Figure 5:**
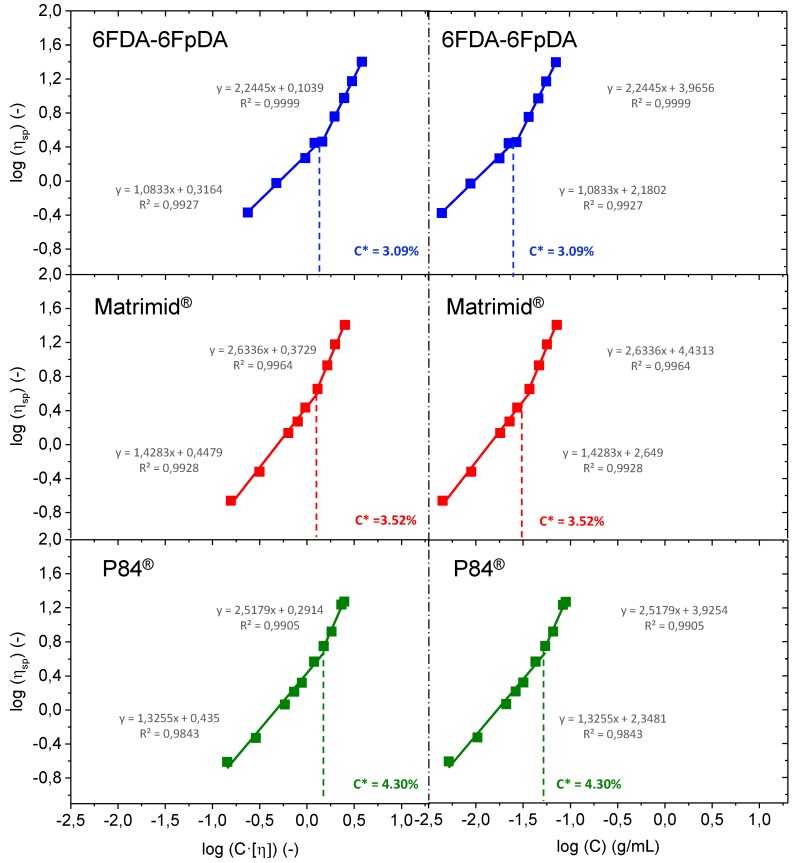
Overlap concentration (C*) for the polymer solutions studied obtained from the representation of log $\eta_{sp}$ as a function of log(C[η] ) (**left** side) and as function of log(C) (**right** side).

Intrinsic viscosity values in mL·g^−1^ were 6FDA-6FpDA ([*η*] = 52.544) > Matrimid^®^ ([*η*] = 34.754) > P84^®^ ([*η*] = 27.249). Once [η] is obtained, the entanglement concentration C* (also called overlap concentration) can be obtained. Plotting the logarithm of the specific viscosity against the logarithm of the coil overlap parameter, which is equal to the product of the concentration (g·mL^−1^) and the intrinsic viscosity (mL·g^−1^), two linear dependencies are obtained. The intersection between these two lines is the so-called overlap concentration [33,34,35]. This representation can be seen in the left part of Figure 5. Overlap concentration can be also calculated by the intersection of the two linear dependencies obtained by the representation of the logarithm of the specific viscosity against the logarithm of the concentration (g·mL^−1^) [36,37,38], as it is plotted in the right part of Figure 5. Values determined by using the two preceding methods were identical. 

The entanglement concentration C*, or overlap concentration, defines the border between the dilute and semi-dilute regions, and is representative of the concentration at which polymer chain entanglement is first observed. In this sense, for concentration values above C*, *C* > C*, the rheological behavior of the system is governed by interactions between multiple coils, rather than by the properties of individual polymer molecules. This results in an increase in polymer interaction, and hence, viscous drag. In this sense, for solutions where *C* < C*, the polymer solution will be in a dilute state, while solutions where *C* > C* will be in a semi-dilute state. In terms of film formation, polymer solutions where *C* > C* will be theoretically more prone to getting continuous polymer layers. 

Membranes for each concentration of polymer solution were obtained, in order to study coating quality, reproducibility, and to carry out the characterization of the separation properties. The selected concentrations were: 0.5–1–2.5–3–4–5–7.5–8–10 wt. %. After the coating process and solvent evaporation at ambient conditions, the membranes were treated under vacuum at 200 °C for 12 h. The temperature of 200 °C was selected in order to ensure that all the solvent used during the dip-coating process is evaporated, and as well as to accelerate the initial aging process in the polymer, which leads to experimental results that are reproducible both in time (samples can be measured at different times after membrane preparation) and for different samples [39]. 

**Figure 6:**
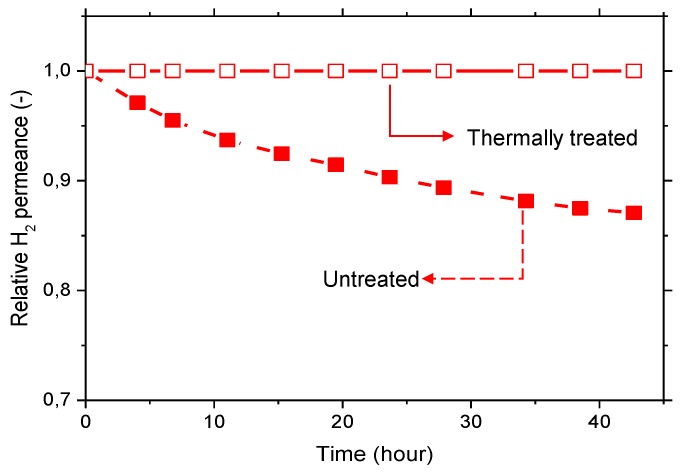
Relative H_2_ permeance at room temperature stabilization test. For untreated and thermally treated 6FDA-6FpDA membrane (5 wt. % polymer solution) supported on alumina Anodisc.

The aging effect is more pronounced for polymers with higher free volume, i.e., the more permeable polymers, such as 6FDA-6FpDA. The origin of the aging effect is related to the relaxation processes that occur in glassy polymers. After the membrane casting, the solvent is continuously evaporating, and this evaporation changes and relaxes, continuously, the polymer conformation. Due to this relaxation, the polymer chains are “arriving” to the low energy level, and the fractional free volume is reduced, leading to a less permeable material [40]. This situation is fostered by thermal treatment at elevated temperatures, leading to polymers with more stable separation properties. The effect of selective layer aging is graphically demonstrated in Figure 6, where the normalized H_2_ permeance over time for 6FDA-6FpDA membranes, not treated and thermally treated in a vacuum oven, are shown. While the untreated membrane showed a decrease of the H_2_ permeance over time, the thermally treated sample presented a constant permeance value. This indicates that, after thermal treatment at 200 °C, the permeation properties are much more stable with time. Therefore, all the films were thermally treated at 200 °C under vacuum for 12 h before further characterization.

**Figure 7:**
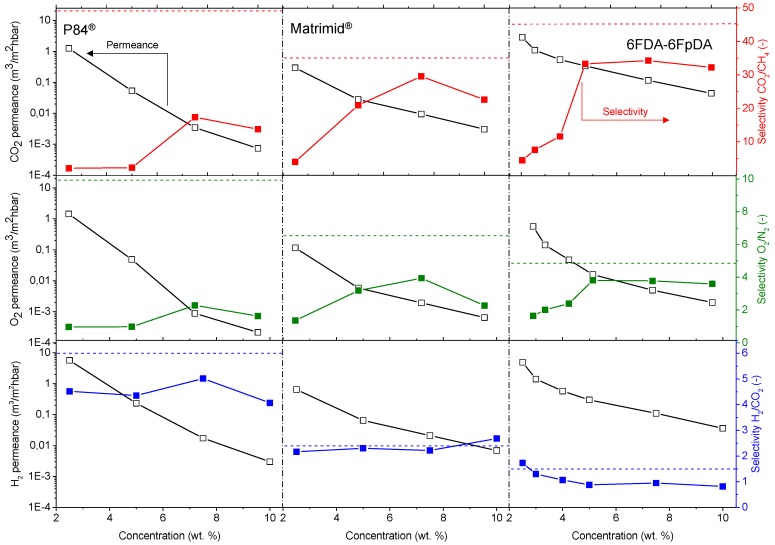
Separation properties of the polymers as a function of the solution concentration at 30 °C. Dashed lines refer to the ideal selectivity for each polymer and gas pair. Permeance values (left-y axis) are plotted in logarithmic scale.

Permeance values and ideal selectivities for the thin film polymer layers were characterized by using the pressure increase facility. CO_2_ and O_2_ permeance, as well as CO_2_/CH_4_ and O_2_/N_2_ selectivities were studied, in order to assess the quality of the polymer layer. On the other hand, H_2_ permeance and H_2_/CO_2_ selectivities were also analyzed for the future applications mentioned in the introduction. Permeances and selectivities obtained as a function of the polymer concentration for P84^®^, Matrimid^®^, and 6FDA-6FpDA, are shown in Figure 7. For all the polymers, permeance values decrease as the polymer concentration in the solution increases. For comparison, the ideal selectivity for each polymer and gas pair was plotted (dashed lines). In this respect, experimental selectivity values closer to the ideal selectivities (thick polymer membranes) mean lower defect concentration in the thin polymeric membranes.

The permeability of the polymer and the permeance of membranes with the same selective layer thickness is related with the polymer FFV: 0.19 for 6FDA [41], 0.17 for Matrimid^®^ [42], and 0.14 for P84^®^ [23]. In this sense, P84^®^ was less permeable than Matrimid^®^, and both membranes were less permeable than 6FDA-6FpDA. Regarding selectivity values, selectivity rises with increasing polymer concentration, generally at the cost of permeance. Solutions with higher polymer concentrations result in thicker layers and lower defect concentration in the thin films. 

The polymers present an ideal CO_2_/CH_4_ selectivity between 35 and 50 for thick films, as shown in Table 1. It is possible to see an evolution of the selectivity for the TFCMs supported by porous alumina for all three polymers. The most permeable polymer, 6FDA-6FpDA, showed CO_2_/CH_4_ selectivity values close to the ideal value obtained for thick films; and the less permeable polymer, P84^®^, showed bigger differences between CO_2_/CH_4_ thick film and TCFM selectivities. As for the permeance, the differences between ideal selectivity for these ceramic-supported polymer membranes and the free-standing thick films were related to the FFV. Indeed, P84^®^ showed bigger differences than Matrimid^®^, and both more differences than 6FDA-6FpDA. 

The highest CO_2_/CH_4_ selectivity value for the ceramic-supported thin film Matrimid^®^ was around 30, which is close to the reported ideal selectivity for integral asymmetric hollow fiber membrane, α(CO_2_/CH_4_) = 33 [43], and similar to the selectivity for the same gas pair in flat sheet integral asymmetric membranes, 30 [44]. For the P84^®^ ceramic-supported thin film, the reported ideal CO_2_/CH_4_ selectivity value for thin film hollow fiber is 12 [43,45], lower than 16, which is the ideal CO_2_/CH_4_ selectivity obtained in this work. For the 6FDA-6FpDA, the ideal CO_2_/CH_4_ selectivity, 35, was lower than the ideal selectivity in thick films, 45. One possible explanation may be related to the sharp edges that the ceramic porous support could have. During the dip-coating process, the polymer is changing its state from vey diluted (even at 10% concentration solution) to the solid state, and evolves to gel state when polymer molecules are already entangled on top of the support. It has been previously reported that in thin films, the free volume is smaller than in thick films, which leads to restrictions in penetrant molecule transport [46]. Despite the outstanding separation properties of the 6FDA-6FpDA polymer, according to our knowledge, thin films out of this polymer in any configuration, i.e., supported by porous ceramics or polymers, were not studied so far. In general, the selectivity values obtained in this work for the ceramic-supported thin films and the reported values for the same materials as a thin film are very similar, which confirms that the casting method was successful. 

Exactly the same behavior in terms of permeance and selectivity was found for the gas pair O_2_/N_2_. Thin films present lower selectivity values than thick films, especially in the case of lowly permeable but highly selective materials. Two main reasons can explain this effect. Firstly, the presence of defects is more likely in thin films, which, eventually, can reduce the selectivity. The second reason is related to their low fluxes through the membrane. When the flux or permeability is very low, the error inherent to the measurement is big, where small differences in permeability could give rise to significant differences in selectivity. The selectivity values for the thin films closest to the ideal selectivity values in thick films were found for a polymer concentration of 7.5 wt % in all the cases. Therefore, membranes obtained from polymer concentration solutions above the entanglement concentration (*C* > C*) are defect-free. 

In case of H_2_ permeance, 6FDA-6FpDA shows the highest values, followed by Matrimid^®^, and finally, P84^®^. For the H_2_/CO_2_ selectivity values, all the polymers present values close to the ideal selectivity for thick films. Considering Figure 7, the minimum concentration required to obtain a continuous and defectless layer can be determined. For P84^®^ and Matrimid^®^, selectivity values for CO_2_/CH_4_ and O_2_/N_2_ start to be acceptable for the 7.5 wt. % concentration solutions. By contrast, in the case of 6FDA-6FpDA, for the same pair of gases, selectivity value becomes approximately constant for solutions with a 5 wt. % concentration. As a conclusion, the minimum concentration value for P84^®^ and Matrimid^®^ is 7.5 wt. % (with a corresponding viscosity of 15 mPa·s) and for 6FDA-6FpDA it is 5 wt. % (viscosity of 5 mPa·s).

**Figure 8:**
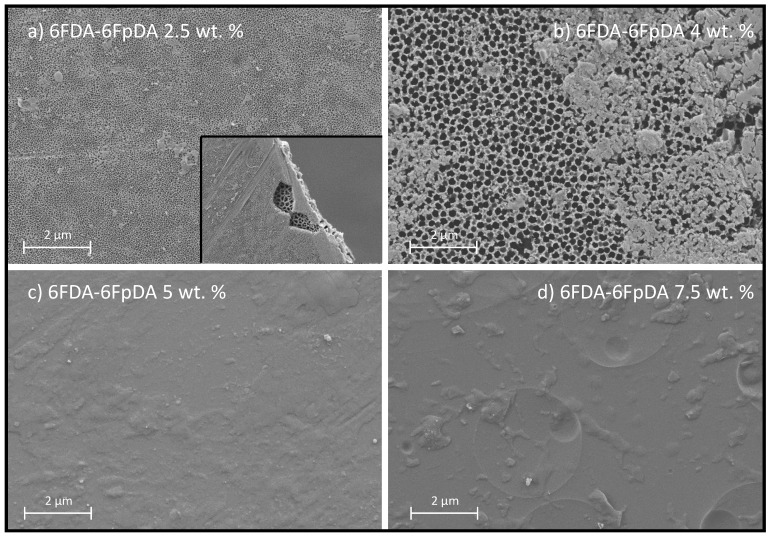
SEM micrographs of 6FDA-6FpDA dip-coated in alumina support with different solution concentrations: C < C* (**a**), C ∼ C*(**b**), and C > C* (**c**,**d**). Inset image in (**a**) corresponds to a magnification where the porous support is observed to be below the polymer layer.

Figure 8 shows the effect of the polymer concentration in the solution on the polymer film formation. Insufficient chain overlap at solution concentrations below or similar to C* led to the formation of discontinuous, if any, polymer layers. For low concentrations, *C* < C*, high penetration of the solvent–polymer system into the pores was observed. For concentrations close to C*, *C* ∼ C*, the polymer formed a film on top of the ceramic support, but still the concentration was not high enough to ensure continuous layer formation. For concentrations above C*, *C* > C*, defect-free polymer layer was repeatedly formed. This observation corresponds with the separation properties presented in Figure 7, where once the polymer concentration in the solution was above the C*, a selective continuous polymeric layer was observed. Note that the pores observed in Figure 8a are smaller than the ones observed in Figure 8b. This fact is due to the polymer penetration into the pores of the substrate that causes the filling of the alumina pores without producing a dense layer. This can be checked in the inset of Figure 8a, where pores formed by the polymer are distinguished from the substrate pores. On the contrary, when *C* = C* (Figure 8b), the polymer is placed on the substrate, and the pores are not filled with polymer.

**Figure 9:**
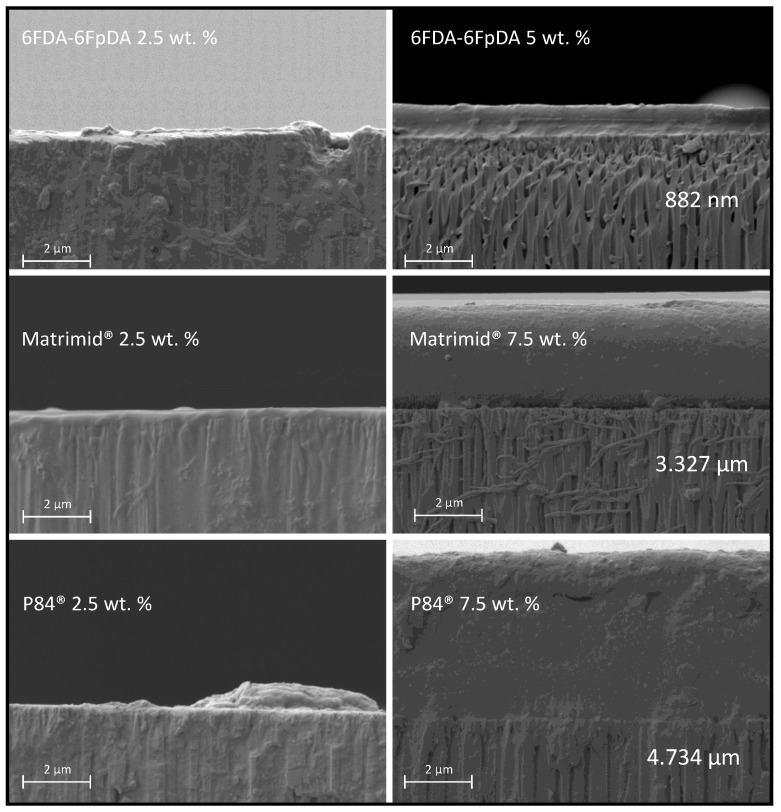
Cross-section SEM micrographs for the polymers deposited with two different solution concentrations: *C* < C* (2.5 wt. %) and *C* > C* (5 wt. % for the 6FDA-6FpDA and 7.5 wt. % for P84^®^ and Matrimid^®^).

SEM micrographs of the cross-section of the prepared membranes gave information on the penetration of the polymer into the pores, thickness of the polymer films as a function of the polymer concentration, and the quality of the porous support coating with polymer films. In Figure 9, two different situations are exposed. For samples deposited from polymer solution, concentrations in the dilute region, *C* < C* (2.5 wt. % in Figure 9), polymer penetrated into the pores of the support due to the lower interaction between the polymer chains. For samples deposited from polymer solution concentrations in the semi-dilute region, *C* > C* (5 and 7.5 wt. % in Figure 9), the penetration of the solution into the porous structure was much lower, and a continuous and defect-free polymeric layer was formed on top of the ceramic support. 

**Figure 10:**
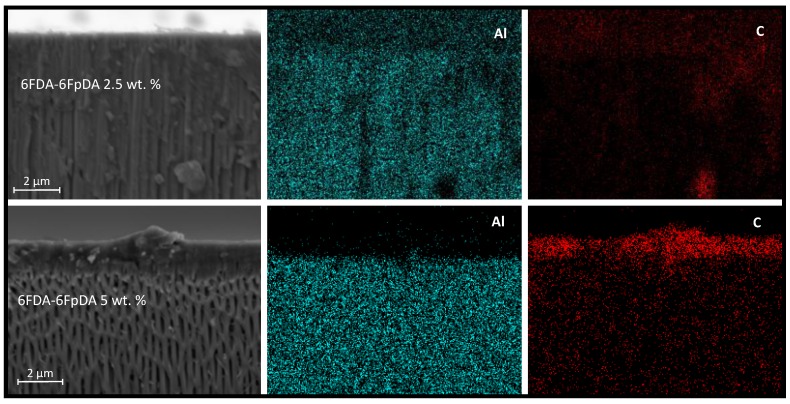
Cross-section micrograph and EDX analysis of 6FDA-6FpDA 2.5 wt. % (**upper** part) and 6FDA-6FpDA 5 wt. % (**lower** part).

In addition, Figure 10 shows EDX analysis (elemental maps for aluminum and carbon) of the cross-section of samples coated with 2.5 wt. % and 5 wt. % of 6FDA-6FpDA. The upper part of the image is related to 2.5 wt. %, where aluminum from the ceramic support presents some blanks that correlate with some penetrated carbon from the polymer solution. Furthermore, the concentration of the penetrated carbon is higher than the deposited carbon on the support. The lower part of the image corresponds to 5 wt. %, where aluminum is homogeneously distributed, and carbon is mainly located on top of the support. Nitrogen and fluorine were also detected by EDX analysis but the low concentration of both hindered the acquisition of accurate elemental maps.

Based on the gas transport data and SEM micrographs, one can again conclude that there is a minimum in polymer solution concentration, *C* > C*, which is needed in order to form a continuous and selective membrane. This minimum concentration, i.e., viscosity, changes depending on the polymer properties. 6FDA-6FpDA, due to its high intrinsic viscosity, gave a defect-free continuous layer at a solution concentration lower than P84^®^ and Matrimid^®^. Indeed, the higher the solution concentration, the closer is selectivity to the ideal value. 

Thickness of the deposited polymer layer generally increases with increasing polymer solution concentration, but, if the concentration was too small, *C* < C*, as shown in Figure 7, membrane selectivity decreases, since the polymer layer is not continuous, as we have shown in Figure 8 for 6FDA-6FpDA. Therefore, the SEM confirmed that a minimum in polymer concentration, *C* > C*, is required to overcome the low compatibility between ceramic and polymeric materials. 

**Figure 11:**
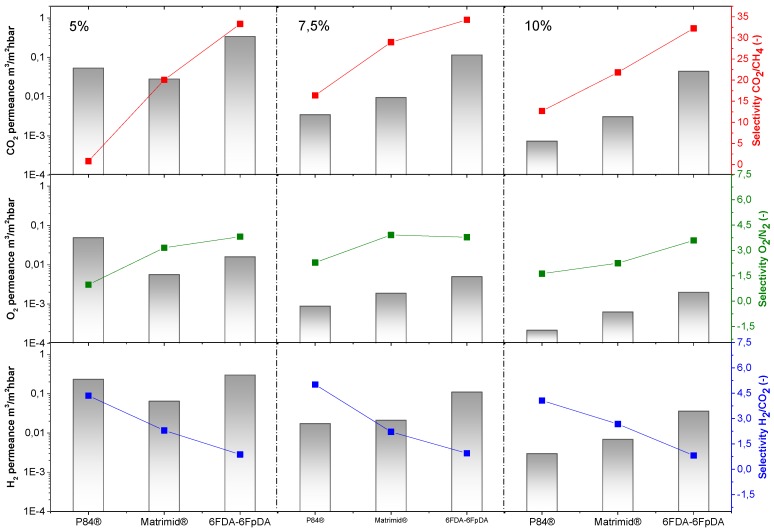
Evolution of the separation properties for the ceramic-supported thin film polymers obtained from 5, 7.5, and 10 wt. % solution for the gas pairs CO_2_/CH_4_, O_2_/N_2_, and H_2_/CO_2_. Permeances (left *y*-axis) are plotted in logarithmic scale at room temperature.

In Figure 11, the separation properties of the ceramic-supported thin film polymers at different concentrations are shown. The evolution of permeance and selectivity for each concentration is depicted, showing that the membrane selectivity remains almost constant for each polymer independent of the concentration, whereas the permeance decreases with the concentration increase, as was discussed above. The differences between the polymers can also be compared, which have to be directly related to their FFV. Therefore, P84^®^ is expected to have the lowest permeances. However, it can be seen that, at the concentration of 5 wt. %, P84^®^ is faster than Matrimid^®^, and in the case of O_2_, even faster than the 6FDA-6FpDA polymer. 

At the same time, the selectivity for this film was very low, and much lower than the selectivities for the films obtained from solutions that were more concentrated. This could be caused by bad formation of the continuous layer on top of the membrane. In Figure 1, the molecular weight of the three polymers is given. The molecular weight of 6FDA-6FpDA is 3.2 times larger than that of P84^®^, and 3.5 larger than that of Matrimid^®^. A higher molecular weight will increment the viscosity of the polymer solution for the same polymer concentration; hence, the formation of a continuous film will be favored. For the rest of the materials and concentrations, the results were as expected, which again confirms the successful deposition of the thin film polymer layer on top of the ceramic support.

The use of ceramic supports and high thermally resistance polymers opens the possibility of the application of polymer TFCMs in processes that occur at elevated temperatures, e.g., water gas shift reactors in coal gasification process. The process requires a final H_2_ purification step, because hydrogen production increases with CO_2_ co-production [47]. Process temperature can vary depending on the plant and other process conditions, but generally the range is between 200 and 500 °C [48,49]. Therefore, the separation properties as a function of temperature were studied to assess the suitability of these materials for operation at higher temperatures.

**Figure 12:**
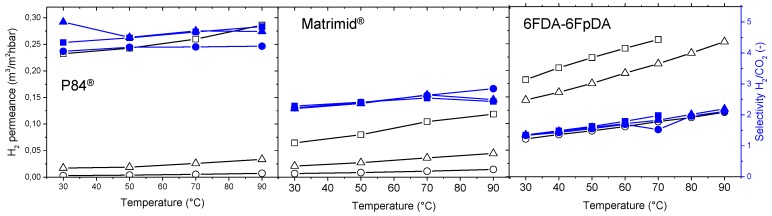
Separation properties of the ceramic-supported thin film polymers for the H_2_/CO_2_ separation as a function of the temperature and concentration of the polymer solution. Unfilled symbols represent the permeance (in the left *y*-axis) and filled symbols represent selectivity (in the right *y*-axis). Squares represent 5% solution, triangles 7.5%, and circles 10%.

H_2_ permeance and H_2_/CO_2_ selectivity were measured at different temperatures, from 30 to 90 °C, as can be seen in Figure 12. Both permeance and selectivity increased with temperature. Depending on the FFV of the polymer, the final selectivity and permeance varied. It can be ascertained that, the lower FFV, the higher the permeability coefficient, and therefore, H_2_ permeability coefficients follow the order P84^®^ < Matrimid^®^ < 6FDA-6FpDA. The film thickness is affecting the permeance values, where higher polymer solution concentrations lead to thicker membranes, and hence, lower permeances. In the case of the H_2_/CO_2_ selectivity, the highly permeable 6FDA-6FpDA presented the lowest selectivity, whereas the low-permeable P84^®^ showed the highest selectivity. While the permeance is affected by the thickness, no significant differences were found for the selectivity. A thinner polymer layer is always preferred, since permeance values will be higher than for thicker polymer layers, if one does not take into consideration the concentration polarization effect, which becomes more important as permeance values increase. In addition, no significant differences in selectivity can be found between thin and thick layers.

The dependence of gas permeance on temperature can be expressed in terms of an Arrhenius-type relationship. It considers the transport of the gas molecules through a membrane as a thermally activated process [50]. The activation energies *E_A_* (Table 3) were calculated from the “pressure increase” experimental results obtained in the range 30–80 °C. Activation energy values depend on the polymer nature, and, in our specific case, are smaller for the polymers with higher FFV [51]. The differences between the E_A_ values for different gases indicate how the selectivity will develop with changing temperature. CO_2_ transport through polymers is strongly dependent on the solubility coefficient, which decreases with increasing temperature. In case of the studied polymers, the change of the diffusion coefficient with the temperature is more significant (in the case of CO_2_, for example) than change of the solubility coefficient. The resulting activation energy of permeability and, consequently, of CO_2_ permeance, is positive. However, since the E_A_ value for CO_2_ is significantly lower than that of H_2_, the selectivity H_2_/CO_2_ increases with temperature rise, opposite to e.g., O_2_/N_2_ selectivity.

**Figure 13:**
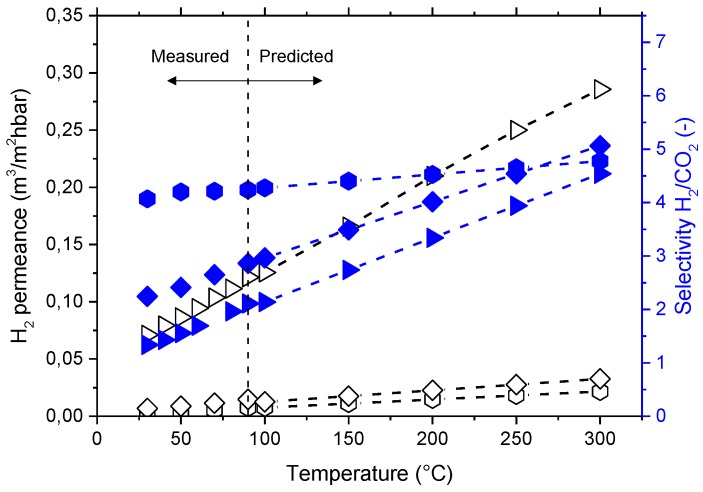
Evolution and prediction of the permeance and selectivity as a function of the process temperature conditions for 10% concentration of polymer. Open symbols represent the permeance (on the left *y*-axis) and filled symbols represent selectivity (on the right *y*-axis). Hexagons represent P84^®^, diamonds Matrimid^®^, and triangles 6FDA-6FpDA.

Figure 13 shows the measured (from 30 °C to 90 °C) and predicted (up to 300 °C) H_2_ permeance and H_2_/CO_2_ selectivity. Membranes will require crosslinking treatment in order to increase their T_g_ and favor their mechanical and chemical properties at temperatures above 300 °C. The H_2_/CO_2_ selectivity of the investigated membranes does only slightly exceed the Knudsen selectivity of 4.69. However, ideal Knudsen flow would require a situation where no convective, non-selective contribution to the flow through a porous membrane would contribute to permeation; this situation is difficult to achieve. The dense, polymeric membrane layer prevents any possibility of convective flow. Furthermore, dense polymer layers may allow (i) the controlled transport of other gaseous components and water through the membrane, and (ii) an additional degree of freedom in process design, i.e., the selection of feed and permeate pressure without considering any selective transport in a pore system.

Summarizing, thin film ceramic-supported membranes are advantageous for H_2_ separation at elevated temperatures, due to their relevant chemical, mechanical, and transport properties up to 300 °C. 

## 4. Conclusions

A technology for the tailored design of selective ceramic-supported thin polymer films was developed. The influence of the polymer solution used in the dip-coating process and its effect in the separation properties was studied. This approach was tested for three different polyimides, P84^®^, Matrimid^®^, and 6FDA-6FpDA. Different polymer solutions were prepared with different concentrations for all the polymers: 0.5–1–2.5–3–4–5–7.5–8–10 wt. %. The deposited thickness was affected by the polymer concentration in the solution. By adjustment of the conditions, defect-free thin films of less than 1 µm thickness were successfully deposited on porous alumina supports for all the studied polymers. 

The influence of the polymer concentration on the separation properties of the materials was thoroughly characterized. By increasing the concentration, thicker polymer layers were achieved, and consequently, lower permeances for all tested gases were measured. In order to test the quality of the polymer deposition, a comparison between the selectivity factors of thick and thin films was carried out. The value for the entanglement concentration (C*) for each polymer under consideration was obtained from the solution viscosity study. The C* values were 3.09 wt. % for the 6FDA-6FpDA, 3.52 wt. % for Matrimid^®^, and 4.30 wt. % for P84^®^. The order for intrinsic viscosity values in mL·g^−1^ were 6FDA-6FpDA (52.544) > Matrimid^®^ (34.754) > P84^®^ (27.249). For the low molecular weight polymers, P84^®^ and Matrimid^®^, highly viscous solutions were needed, around 15 mPa·s, while for the high molecular weight 6FDA-6FpDA polymer, less than 5 mPa·s was sufficient to get a continuous defect-free polymer layer. 

The polymers were selected for their differences in permeability, where the evolution of the FFV is P84^®^ < Matrimid^®^ < 6FDA-6FpDA. The same tendency was found for the ceramic-supported thin film polymers. The effect of the temperature on the separation properties was studied. As it can be expected, the permeance for all the gases tested increased with the temperature, but the increase of the H_2_ permeance was higher than for the rest of the gases, included CO_2_. Therefore, an increase of the H_2_/CO_2_ selectivity with temperature was observed for all the polymers and compositions. The prediction of the properties at elevated temperatures showed that the selectivity raises with decreasing FFV: P84^®^ > Matrimid^®^ > 6FDA-6FpDA. 

This study shows the potential of this approach, in order to apply polymeric membranes for processes at elevated temperatures. Nevertheless, more efforts are necessary in order to see the real prospect of this approach:

1. Study of the system under conditions closer to the real application in terms of temperature (200–250 °C) and pressure (10–20 bar).
2. Improvement of the casting conditions in terms of thinner layers (<200–300 nm), lowering the entanglement viscosity (by very high molecular weight polymers or by crosslinking reactions), and polymers soluble in highly volatile solvents (CH_3_Cl or THF).
3. Study of the influence of the pore size and composition of the ceramic support in the polymer layer formation (in order to reduce polymer penetration into the pores).
4. Transferring all the knowledge to the typical tubular ceramic membranes that are finally placed in the process.

## Figures and Tables

**Table 1:** Ideal permeability and selectivity for the polymers studied in this work as a thick film at room temperature.

	Permeability (Barrer)			Selectivity (-)		
	H_2_	O_2_	CO_2_	H_2_/CO_2_	O_2_/N_2_	CO_2_/CH_4_
P84^®^ [23]	7.2	0.24	1.2	6.0	10.0	50.0
Matrimid^®^ [24]	17.5	1.5	7.3	2.4	6.6	34.7
6FDA-6FpDA	93.3	12.9	63.8	1.5	4.8	45.6

**Table 2:** Viscosity nomenclature.

Dynamic Viscosity (Solution Viscosity)	η
Solvent viscosity	$\eta_{s}$
Concentration of the solution	C
Relative viscosity	$\eta_{rel}=\frac{\eta }{\eta_{s}}$
Specific viscosity	$\eta_{sp}=\frac{\eta -\eta_{s}}{\eta_{s}}=\eta_{rel}-1$
Reduced viscosity	$\eta_{red}=\frac{\eta_{sp}}{C}$
Inherent viscosity	$\eta_{inh}=\frac{ln\eta_{rel}}{C}$
Intrinsic viscosity	$\left[\eta \right]=\underset{C\to 0}{\lim}\left(\eta_{red}\right)=\underset{C\to 0}{\lim}\left(\eta_{inh}\right)$

**Table 3:** Activation energies of permeance for the different gases for thin film composite membranes (TFCMs) prepared using 10 wt. % polymer solutions.

	Activation Energy (*E_A_*) (KJ·mol^−1^)				
	CO_2_	O_2_	H_2_	CH_4_	N_2_
**P84^®^**	13.9	20.74	13.66	32.29	21.53
**Matrimid^®^**	7.82	19.80	11.58	33.42	28.60
**6FDA-6FpDA**	1.63	7.83	8.07	21.60	19.75
